# Nucleic Acid Armor: Fortifying RNA Therapeutics through Delivery and Targeting Innovations for Immunotherapy

**DOI:** 10.3390/ijms25168888

**Published:** 2024-08-15

**Authors:** Yi Jiang, Bolong Jiang, Zhenru Wang, Yuxi Li, James Chung Wai Cheung, Bohan Yin, Siu Hong Dexter Wong

**Affiliations:** 1 School of Medicine and Pharmacy, The Ocean University of China, Qingdao 266100, China; jiangyi@stu.ouc.edu.cn (Y.J.); jiangbolong@stu.ouc.edu.cn (B.J.); liyuyuxi@163.com (Y.L.); 2 Medical College, Jining Medical University, Jining 272000, China; zhenru_wang@163.com; 3 Department of Biomedical Engineering, The Hong Kong Polytechnic University, Kowloon, Hong Kong 999077, China; james.chungwai.cheung@polyu.edu.hk; 4 Laboratory for Marine Drugs and Bioproducts, Qingdao Marine Science and Technology Center, Qingdao 266237, China

**Keywords:** RNA drugs, nucleic acid delivery, nanotechnology, immunotherapy, antibody–oligonucleotide conjugates

## Abstract

RNA is a promising nucleic acid-based biomolecule for various treatments because of its high efficacy, low toxicity, and the tremendous availability of targeting sequences. Nevertheless, RNA shows instability and has a short half-life in physiological environments such as the bloodstream in the presence of RNAase. Therefore, developing reliable delivery strategies is important for targeting disease sites and maximizing the therapeutic effect of RNA drugs, particularly in the field of immunotherapy. In this mini-review, we highlight two major approaches: (1) delivery vehicles and (2) chemical modifications. Recent advances in delivery vehicles employ nanotechnologies such as lipid-based nanoparticles, viral vectors, and inorganic nanocarriers to precisely target specific cell types to facilitate RNA cellular entry. On the other hand, chemical modification utilizes the alteration of RNA structures via the addition of covalent bonds such as N-acetylgalactosamine or antibodies (antibody–oligonucleotide conjugates) to target specific receptors of cells. The pros and cons of these technologies are enlisted in this review. We aim to review nucleic acid drugs, their delivery systems, targeting strategies, and related chemical modifications. Finally, we express our perspective on the potential combination of RNA-based click chemistry with adoptive cell therapy (e.g., B cells or T cells) to address the issues of short duration and short half-life associated with antibody–oligonucleotide conjugate drugs.

## 1. Introduction

With the progressive advancement of research and therapeutic approaches in the field of medicine, there has been a growing awareness of safety concerns and drug resistance related to conventional treatment methods. Simultaneously, the efficacy of traditional small molecule drugs and immunotherapy strategies in treating various diseases has been considerably impeded due to the limited understanding or unavailability of target protein mechanisms and receptors [1].

RNA is a crucial biomolecule involved in protein synthesis within most living organisms’ bodies. Specifically, messenger RNA (mRNA) is a type of ribonucleic acid responsible for transcribing genetic information from DNA into the necessary information for protein synthesis. In cellular processes, genetic information is initially transcribed into mRNA molecules through the transcription process. Subsequently, these mRNA molecules are translated into proteins with the aid of ribosomes. In 1990, Jon Wolff at the University of Wisconsin achieved a groundbreaking breakthrough by demonstrating that the direct injection of mRNA into animals resulted in protein expression [2]. This pivotal finding marked the advent of a new era in nucleic acid therapeutics. Based on their intended purpose, these encoded proteins can be classified as therapeutic proteins that enhance human immune response or pathogenic proteins that serve as antigens, effectively stimulating the immune system [3]. As a result, mRNA-based drugs are capable of targeting a vast range of human diseases, such as cancer and infection by pathogens [4].

**Figure 1:**
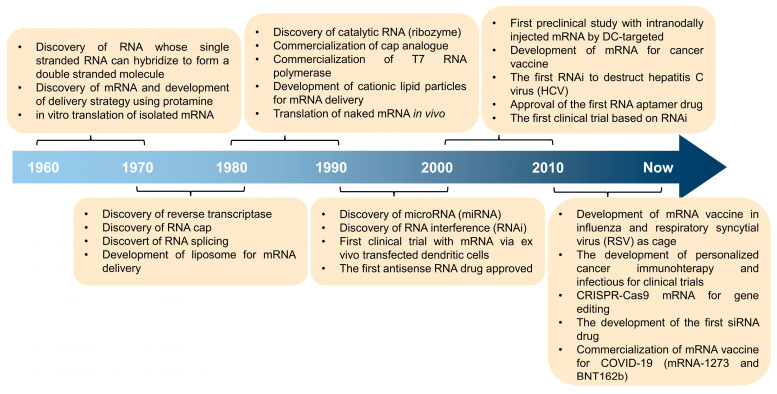
The timeline for RNA-based therapeutic development for various diseases and immunotherapy.

A class of nucleic acid drugs known as RNA interference (RNAi) drugs emerged, including micro-RNA (miRNA), antisense oligonucleotide (ASO), and small interference RNA (siRNA) [5]. In contrast to mRNA, RNAi primarily functions by suppressing gene expression, thereby preventing the production of essential proteins and potentially upregulating the loss of functional phenotypes, cell death, or impaired proliferation [6]. ASOs exert their biological effects through three distinct mechanisms: mRNA silencing, inhibition of ribosome translation, or modulation of RNA splicing [7]. siRNA is a short-stranded RNA molecule that regulates gene expression by binding to complementary mRNA sequences, leading to mRNA degradation or transcriptional inhibition. miRNA is a small, non-coding RNA molecule found in eukaryotic organisms. It regulates gene expression by partially binding to complementary sequences in the 3’ untranslated region of target mRNA, leading to translational repression or mRNA degradation. Both siRNA and miRNA rely on Dicer enzyme processing or the formation of an RNA-induced silencing complex (RISC) to suppress the translation of target mRNAs [8]. The main distinction lies in the nature of siRNA and miRNA. siRNA is a synthetic double-stranded RNA capable of binding to any segment of mRNA, whereas miRNA is an endogenous single-stranded RNA that functions at a specific site. Furthermore, there are nucleic acids with unique structures, such as aptamers (short, single-stranded DNA or RNA molecules), which possess the ability to bind to and inactivate specific proteins with high affinity and specificity [9]. These aptamers have shown promising efficacy and hold significant potential for further research and development. siRNAs are double-stranded RNA molecules that are typically 20–25 base pairs in length. They are used to trigger the RNA interference (RNAi) pathway, which can silence the expression of specific genes by degrading the corresponding mRNA [10]. mRNA is a single-stranded RNA molecule that carries the genetic code from the nucleus to the ribosome, where it is translated into proteins. mRNA molecules are significantly larger than siRNAs, typically hundreds to thousands of nucleotides in length [11]. Delivering mRNA can be more challenging than smaller RNAs, as the larger size makes it harder to encapsulate and protect the molecule during delivery. Self-amplifying mRNA (samRNA) is a type of mRNA that contains additional sequences that allow it to self-amplify inside the target cells. This self-amplification can lead to higher levels of protein expression compared to regular mRNA. samRNA is also larger and more complex than regular mRNA, which can make it more difficult to deliver effectively [12]. In summary, siRNAs are smaller and more stable, making them easier to deliver, while mRNA and samRNA are larger and more complex, presenting additional challenges for effective delivery to target cells. Up until now, a large number of RNA therapeutics have been developed for infectious diseases and immunotherapy by improving the stability, translation efficiency, and immune response of the RNA (Figure 1).

## 2. RNA-Based Immunotherapy

Immunotherapy is a cutting-edge approach that harnesses the innate immune system of patients to combat diseases. By enhancing the activity of T cells or disrupting the immune evasion strategies of disease cells or viruses, immunotherapy empowers the immune system to triumph over disease [13]. Conventionally, immunotherapy involves identifying immune checkpoints and developing checkpoint inhibitors for medications [14], such as monoclonal antibodies (mAbs) [15], bispecific antibodies [16], and trispecific antibodies [17]. Alternatively, coupling an antibody and a small molecule drug together can precisely deliver them to target cells and then exert their pharmacological effects, resulting in effective therapeutic outcomes. This concept is known as Antibody–Drug Conjugates (ADCs) [18].

In addition to using antibodies and antigens for binding, RNA-based immunotherapy offers a novel avenue for precise and targeted intervention. For example, using mRNA vaccines to treat COVID-19 is an RNA-based immunotherapy. mRNA is internalized into the cytoplasm under the synergistic effect of the vector, translated into the target antigen in the cytoplasm, and the target antigen enters the internal environment to exert immune efficacy [19]. Meanwhile, Professor William Schief invented the first human HIV vaccine elicited by mRNA, and Moderna discovered a multiclade env-gag VLP mRNA vaccine that can elicit HIV antibodies and reduce the risk of heterologous tier-2 simian–human immunodeficiency virus (SHIV) infection. The above two vaccines utilize mRNA to stimulate immune cells to secrete antibodies and induce the cell’s immune reaction [20].

## 3. RNA Delivery Systems

RNA drugs are not exempt from challenges, including issues of instability, rapid elimination, and notable off-target effects. Furthermore, they face limitations in their ability to precisely target and efficiently enter cells [21]. Therefore, the development of an effective delivery system for RNA drugs can resolve these limitations. Two distinct methods have been devised to bolster the stability of nucleic acid drugs and aid in their targeting and cellular entry: delivery vehicles and chemical modifications.

### 3.1. Delivery Vehicles

**Figure 2:**
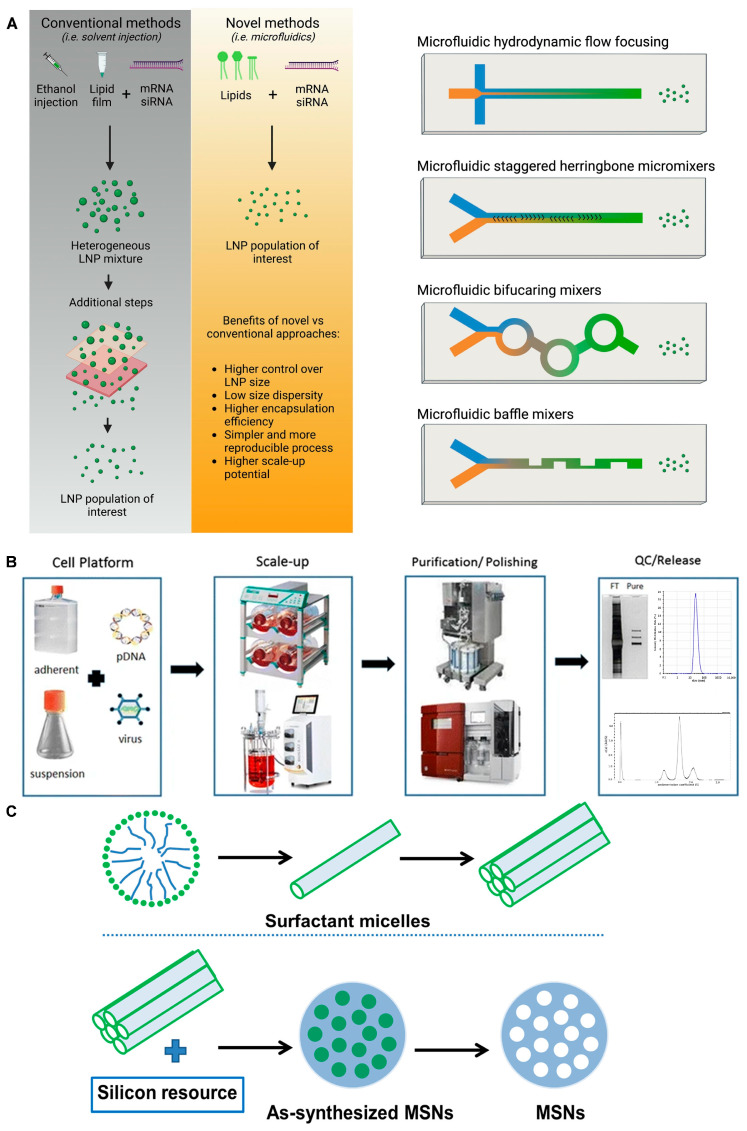
Preparation method of delivery vehicles. (**A**) Workflow of conventional and novel approaches for LNP preparation. Figure is adapted from [22]. (**B**) Overview of adeno-associated virus (AAV) production/purification. Figure is adapted from [23]. (**C**) Schematic diagram showing the preparation of mesoporous silica nanoparticles (MSNs). Figure is adapted from [24].

The delivery vehicle refers to the system or carrier used to transport and deliver the RNA therapeutic to the target cells or tissues. Typically, the RNA is embedded within a substance capable of transporting it to a designated site, enabling it to elicit a therapeutic effect at the specific location. Moreover, the vehicle enhances the stability and transmembrane delivery of RNA without altering its nature. Here, we explore several types of RNA delivery vehicles and their preparation methods: lipid nanocarriers, viral vectors, and inorganic nanocarriers (Figure 2 and Table 1).

### 3.2. Lipid Nanoparticles (LNPs) and Liposomes

Lipid materials, owing to their lipophilic properties, facilitate efficient delivery within the body. Unlike conventional liposomes, which are spherical structures composed of a phospholipid bilayer with hydrophilic interiors housing nucleic acids [38], current lipid nanocarriers exhibit a more focused approach. Liposomes and lipid nanoparticles (LNPs) share similarities in their design, but they differ in their composition and function. Both are lipid-based nanoformulations and are excellent drug delivery vehicles, transporting their cargo within a protective, outer layer of lipids. However, LNPs can take on various forms. LNPs are similar to liposomes, but they are specifically designed to encapsulate a wide range of nucleic acids (RNA and DNA). As a result, LNPs have become the most popular non-viral gene delivery system. Companies such as Exelead develop and manufacture LNPs to encapsulate different types of genetic payloads, including siRNA, mRNA, and saRNA. Traditional liposomes typically have one or more rings of a lipid bilayer surrounding an aqueous pocket, but not all LNPs have a continuous bilayer structure that would qualify them as lipid vesicles or liposomes. Some LNPs assume a micellar structure, encapsulating drug molecules in a non-aqueous core. An additional lipid aids in accelerating the structural transformation of LNPs within cells, facilitating drug release. Meanwhile, the outer layer is often coated with polyethylene glycol (PEG) lipids to reinforce colloidal stability and prevent protein absorption or aggregation in circulation, thus mitigating immune clearance [30]. In addition, delivery systems for RNA delivery via anionic or zwitterionic liposomes have also emerged. Studies by Siddhesh D Patil et al. have shown that the transfection efficiency of some anionic liposomes is similar to that of cationic liposomes, while their toxicity is significantly lower [39]. The preparation process of LNPs can be more conveniently adjusted in size to adapt to more functions under novel methods such as microfluidic (Figure 2A). Both liposomes and LNPs facilitate RNA delivery, and their mechanisms are analogous. Specific microenvironments, such as the tumor microenvironment (TME), show altered structure and permeability of blood vessels that potentially permit preferential accumulation of liposomes and LNPs in the environment for passively targeting therapeutic effects [30].

Three lipid-based delivery systems have been commercialized to date. Among them, Onpattro mainly treats hereditary transthyretin amyloidosis (hATTR). hATTR is mainly caused by the accumulation of TTR proteins due to mutations in the transthyretin (TTR) gene in hepatocytes. Onpattro inhibits mutant TTR expression by silencing cells by silencing LNP−siRNA accumulation in hepatocytes. Clinical data also prove the effectiveness of Onpattro [40]. The other two are used in COVID-19 vaccines [41]. However, these approved RNA therapeutics face challenges related to limited storage duration and stringent transportation requirements. Furthermore, the inclusion of PEG to mitigate immune clearance and enhance stability is associated with increased cytotoxicity [42], rendering the balance between stability and undesirable side effects a contemporary challenge. To address this, various PEG substitutes have emerged to improve the side effects [43], such as poly(glycerols) (PGs), poly(oxazolines) (POX), poly(hydroxypropyl methacrylate) (PHPMA), and poly(2-hydroxyethyl methacrylate) (PHEMA) [38,44].

**Figure 3:**
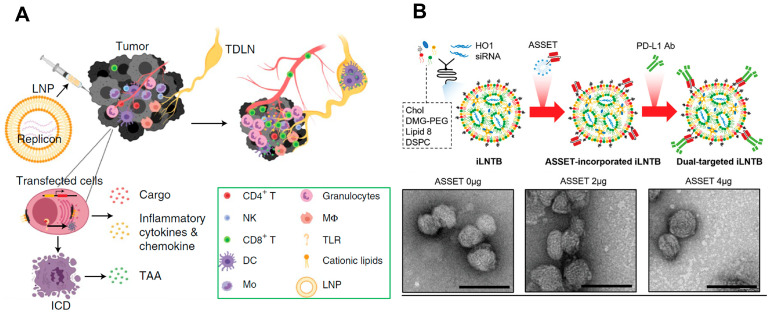
Lipid and liposome-based vehicles for immunotherapy. (**A**) Lipid nanoparticles (LNPs) for promoting immunogenic cancer cell death (ICD), delivery of RNA to stimulate danger sensors in transfected cells, and delivery of RNA-encoded interleukin (IL)-12 fusion protein production in cancer cells for boosting immune cells’ antitumor activities. Figure is adapted from [45]. (**B**) A nanobooster targeting programmed cell death protein 1 (PD-L1) and inhibiting heme oxygenase-1 (HO1) (siRNA) for cancer chemo-immunotherapy. Figure is adapted from [46]. Scale bars indicate a 100 nm scale.

Recently, Weiss et al. reported the development and evaluation of a multifunctional oncolytic nanoparticle system for cancer immunotherapy [45]. They fabricated the LNP using an ionizable lipid (called TT3) that can package and deliver self-replicating RNA (replicons) encoding an interleukin-12 (IL-12) fusion protein (Figure 3A). The TT3 lipid nanoparticles (LNPs) were able to efficiently transfect tumor cells with the replicon, leading to high levels of IL-12 expression, activation of innate immune pathways, and induction of immunogenic cell death (ICD) in the transfected tumor cells. A single intratumoral injection of the LNP replicons encoding IL-12 was able to eliminate large established tumors in several mouse cancer models. This was attributed to the synergistic effects of LNP-mediated ICD, inflammatory cytokine production, and innate immune stimulation. The fusion of IL-12 to the matrix-binding protein lumican helped avoid systemic toxicity by enhancing the retention of IL-12 within the tumor microenvironment. The LNP replicon approach was able to prime systemic antitumor immunity and induce immune memory, enabling the regression of distal untreated tumors.

Chemo-immunotherapy, combining standard chemotherapy with immunotherapy such as immune checkpoint inhibitors (ICIs), is a promising approach for treating various cancers [47]. However, many cancer patients still respond poorly to current chemo-immunotherapy regimens, suggesting the need for novel boosting agents such as nanotherapeutics. The tumor immune microenvironment is often enriched with immunosuppressive tumor-associated macrophages (TAMs) that impede the infiltration of cytotoxic CD8^+^ T cells [48]. Targeting TAMs and overcoming their immunosuppressive effects could help transition “cold” tumors to “hot” tumors that are more responsive to immunotherapy. Heme Oxygenase-1 (HO1) acts as an immunotherapeutic molecule in tumor myeloid cells, in addition to promoting chemoresistance in cancer cells. Inhibiting HO1 could be a promising strategy to boost chemo-immunotherapy, but available HO1 inhibitors have serious adverse effects. Peer et al. reported a tumor myeloid cell and cancer cell dual-target LNP formulation loaded with HO1-inhibiting siRNA (T-iLNTB) (Figure 3B) [46]. T-iLNTB-mediated HO1 inhibition sensitized cancer cells to chemotherapeutics by increasing immunogenic cell death. T-iLNTB also directly reprogrammed tumor myeloid cells, leading to the recruitment of cytotoxic CD8^+^ T cells and “cold-to-hot” tumor transition. T-iLNTB treatment enhanced the efficacy of chemo-immunotherapy in tumor models. In addition, HO1 inhibition also directly affected tumor macrophage differentiation in ex vivo experiments. This work represents a novel therapeutic modality that can boost the efficacy of chemo-immunotherapy by simultaneously targeting cancer cells and immunosuppressive TAMs.

### 3.3. Viral Vectors

**Figure 4:**
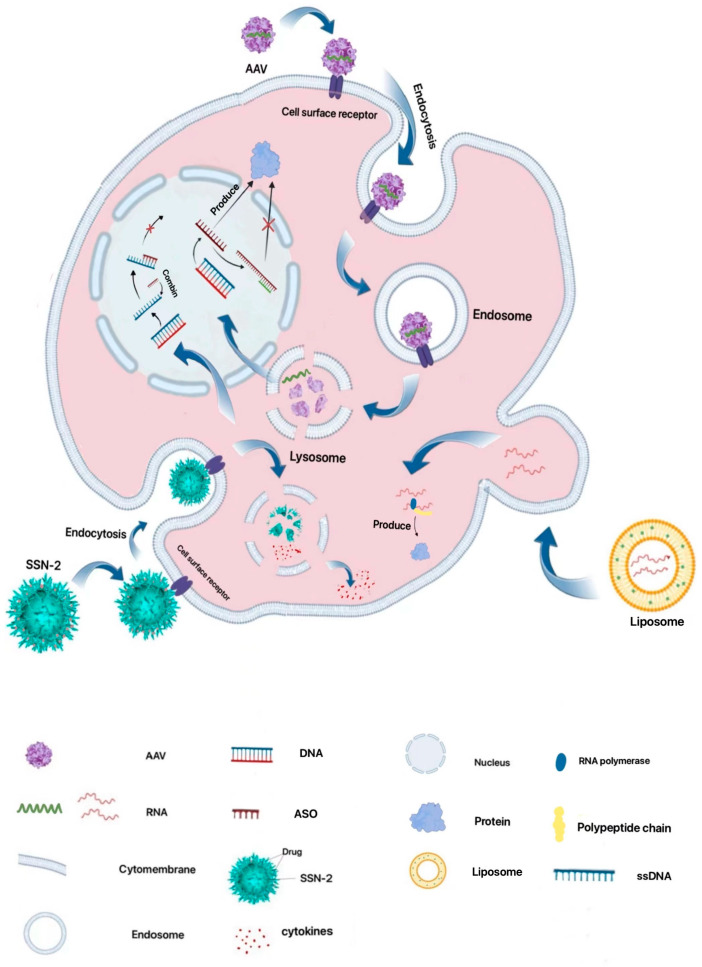
Schematic illustration of various RNA delivery approaches with their cellular entry pathway mechanisms.

Given that viruses invade the body and replicate their genetic material through host cells, researchers have harnessed this principle to design viral vectors for effective RNA delivery into target cells. Currently, prevalent viral vector platforms include lentiviral, adenoviral, and adeno-associated viral (AAV) vectors [49]. While the fundamental principles underlying the infection and replication mechanisms of these viral vector types are analogous, each possesses its unique set of advantages and disadvantages for viral vector-mediated gene delivery, generally involving the following steps. First, the viral vector binds to specific receptors on the target cells, triggering endocytosis. Subsequently, the endosome containing the viral vector disintegrates and escapes, influenced by changes in pH and other factors [50]. This process allows the genetic cargo to enter the nucleus, while the viral capsid protein is hydrolyzed into polypeptides within the cytoplasm (Figure 4).

The genes of the lentivirus integrate seamlessly into the host genome, enabling sustained expression of the delivered genes and prolonged drug efficacy. However, the random nature of this genomic integration necessitates further rigorous research and scrutiny to ensure the safety of lentivirus-mediated gene delivery [51]. Several products based on lentiviral vectors from Bluebird have led to safety incidents caused by the random nature of genomic integration at the clinical stage. For example, one patient was diagnosed with myelodysplastic syndrome, while another patient developed acute myeloid leukemia after receiving LentiGlobin gene therapy [52]. The FDA has therefore shelved its clinical trial protocol for LentiGlobin gene therapy for sickle cell disease (SCD). Therefore, the potential safety concerns of viral vectors cannot be negligible before clinical applications. In contrast, adenoviral vectors possess the capacity to package and deliver larger genetic payloads without integrating into the host genome, allowing for rapid transgene expression. Nonetheless, their expression of foreign genes mediated by adenoviral vectors is generally transient, and they tend to elicit a stronger immune response compared to other viral vector platforms (Figure 2) [31].

Among the prevalent viral vector platforms, AAV is a more commonly utilized vector that stands out for its high safety profile, stemming from its non-integrative nature. While its packaging capacity is limited and the transgene expression kinetics are slower compared to other viral vectors, AAV has shown the potential to extend the duration of therapeutic gene expression to over six months [53]. Furthermore, AAV elicits minimal immune responses and exhibits a low frequency of genomic integration, further enhancing its safety profile. Notably, seven gene therapy products approved by regulatory agencies, such as the Food and Drug Administration (FDA, U.S.), European Medicines Agency (EMA), and Pharmaceuticals and Medical Devices Agency (PMDA, Japan), have employed AAV-like viral vectors as the delivery platform (Figure 2B) [23], used for the treatment of rare diseases, hemophilia, and ophthalmic diseases. Despite the advantages of viral vectors, it is well-established that the viral capsid proteins are recognized as antigenic epitopes, triggering host immune responses and leading to clearance of the viral particles. This immune recognition and clearance significantly diminish the therapeutic efficacy of viral vectors upon subsequent administrations, thus limiting the long-term application of these delivery platforms [54].

**Figure 5:**
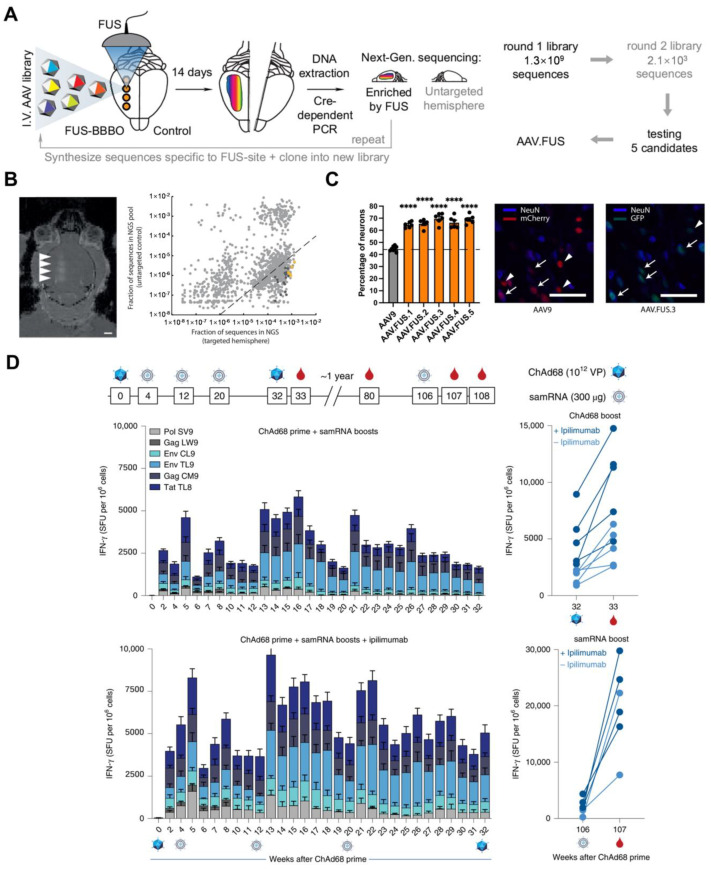
Virus-based RNA vectors for immunotherapy. (**A**) High-throughput in vivo selection to engineer new adeno-associated virus vectors specifically designed for local neuronal transduction at the site of focused ultrasound blood–brain barrier opening (FUS-BBBO); (**B**) visualization of the copy number in each hemisphere. Yellow dots represent 5 clones (AAV.FUS.1−5) selected for low-throughput testing.; (**C**) all AAV.FUS candidates had higher neuronal tropism. Black dots represent data from 3 male and 3 female mice for corresponding serotypes. Image AAV9 represents the transduce both neurons (blue, NeuN staining, example neurons designated by an arrow) and non-neuronal cells (example non-neuronal cells designated by an arrowhead). Image AAV.FUS.3 represents more of the cells transduced with AAV.FUS (green) are neurons (example neurons designated by an arrow), rather than non-neuronal cells (example cell designated by an arrowhead). Figures are adapted from [55]. The scale bars are 50 μm. (**** *p* < 0.0001, ANOVA) (**D**) Heterologous prime-boost vaccination, using the chimpanzee adenovirus ChAd68 followed by self-amplifying RNA (samRNA) to induce broad and long-lasting CD8^+^ T cell responses in non-human primates. Figure is adapted from [56].

Szablowski et al. recently presented a novel approach to improving gene delivery to the brain using focused ultrasound blood–brain barrier opening (FUS-BBBO) and engineered (AAV) vectors (Figure 5A) [55]. Targeted gene delivery to the brain is a major challenge in neuroscience research and disease treatment. Traditional approaches such as direct brain injections are invasive, while systemic or intrathecal delivery lacks spatial precision. FUS-BBBO provides a noninvasive method to temporarily open the blood–brain barrier and allow the passage of AAVs from the bloodstream into specific brain regions. However, this method shows low transduction efficiency at the FUS-BBBO site and leads to the undesirable transduction of peripheral organs due to AAV entry through the leaky BBB. Thus, the authors performed high-throughput in vivo screening to identify AAV capsid mutations that enhance transduction at the FUS-BBBO site while reducing off-target peripheral transduction. The Screened AAV capsid mutations significantly improved on-target transduction efficiency (>10-fold) compared to wild-type AAVs, with increased neuronal tropism (Figure 5C) and reduced off-target transduction in peripheral organs (Figure 5B). This work overcomes the limitations of current noninvasive brain gene delivery approaches, with the potential to advance neuroscience research and the treatment of brain disorders.

In another study, Jooss et al. presented an ongoing phase 1/2 clinical trial (GRANITE, NCT03639714) evaluating the safety, tolerability, and immunogenicity of an individualized, heterologous vaccine regimen consisting of a chimpanzee adenovirus (ChAd68) vector and a self-amplifying mRNA (samRNA) vector, in combination with the checkpoint inhibitors nivolumab and ipilimumab, for the treatment of advanced metastatic solid tumors (Figure 5D) [56]. The vaccine targets patient-specific tumor neoantigens and is designed to prime and expand tumor-specific T-cell responses, which are critical for tumor control in response to checkpoint inhibitor therapy. In non-human primate studies, the heterologous vaccine regimen elicited potent and long-lasting T cell responses against simian immunodeficiency virus (SIV) model antigens, and subcutaneous administration of anti-CTLA-4 antibodies enhanced the vaccine-induced immune responses. In the ongoing phase 1/2 clinical trial, the individualized vaccine regimen was found to be safe and well-tolerated, with no dose-limiting toxicities. The recommended phase 2 dose was determined to be 10^12^ viral particles of ChAd68 and 30 μg of samRNA. The vaccine manufacturing process was feasible, and vaccination induced long-lasting neoantigen-specific CD8^+^ T cell responses in patients. Several patients with microsatellite-stable colorectal cancer (MSS-CRC) showed improved overall survival, and exploratory biomarker analyses revealed decreased circulating tumor DNA (ctDNA) in patients with prolonged survival. Although the small sample size limits statistical and translational analyses, the increased overall survival observed in MSS-CRC patients warrants further investigation in larger randomized studies. Overall, the results suggest that the individualized, heterologous vaccine regimen, in combination with checkpoint inhibitor therapy, has the potential to generate robust and durable T-cell responses against tumor neoantigens, leading to improved clinical outcomes for patients with advanced metastatic solid tumors.

### 3.4. Inorganic/Polymer Nanomaterial-Based RNA Delivery Vehicles

In addition to the lipid- and viral vector-based carriers, a wide range of inorganic and polymer-based nanomaterials have garnered substantial attention as delivery systems for RNA-based therapeutics [57]. These nanoparticles offer distinctive advantages in safeguarding the RNA cargo, effectively shielding it from degradation by RNase enzymes through mechanisms such as steric hindrance and electrostatic repulsion [58]. Through precise control over their physicochemical properties, surface area, and modifications (e.g., incorporation of quaternary ammonium groups or cationic lipid membrane coatings), these nanoparticle-based delivery systems can be tailored and optimized for the effective delivery of RNA-based drug payloads. Cationic polymers, such as polyethyleneimine (PEI), chitosan, and polypeptides, can electrostatically complex with negatively charged RNA molecules to form nanoparticles [59,60]. These polymer-based nanoparticles can protect RNA from degradation, facilitate cellular uptake, and promote endosomal escape. Biodegradable and biocompatible polymers, such as poly(lactic-co-glycolic acid) (PLGA) and poly(ε-caprolactone) (PCL), have also been used to develop RNA delivery vehicles [61].

Among the diverse array of inorganic nanoparticle platforms, mesoporous silica nanoparticles (MSNs or MSNPs) have emerged as one of the most promising delivery systems for RNA-based therapeutics. However, given the relatively short research history of MSNs and their safety concerns, this technology is still in the experimental phase and has yet to reach practical clinical applications. In contrast to LNP systems, MSNs provide greater flexibility for personalization and optimization by allowing for the adjustment of key parameters such as mesoporous pore size and surface functional group modifications (Figure 2C). The interfacial design of MSNs can be ingenious and multifaceted. On one hand, the pores within the MSN structure serve as the cage, enabling them to effectively encapsulate and transport RNA-based drugs. RNA can be unstable in the complex physiological fluid. The surface of MSNs can be functionalized with a positive charge that electrostatically pre-adsorbs the negatively charged RNA for loading. Furthermore, once the MSNs are internalized by target cells, the encapsulated RNA can be efficiently adsorbed and subsequently released into the intracellular compartments with decreased pH. In addition, the silanol groups of the MSN surface can be further modified with various targeting ligands to ascertain its targeting ability [24]. As a recent example, Wang et al. recently developed a novel virus-mimicking spiked silica nanoparticle (SSN). By precisely controlling the binding duration between 3-aminophenol and formaldehyde and the silica core with optimized spike lengths, a positive surface charge was imparted through the chemical modification of amino groups. This unique SSN architecture efficiently adsorbed and sequestered RNA within the open spaces between the protruding spikes, thereby creating a solid–liquid interface that enhanced the stability of the adsorbed RNA cargo. The SSNs subsequently released the RNA payload inside the cells while effectively minimizing cytotoxicity, presenting promising applications for the treatment of cancer [62].

**Figure 6:**
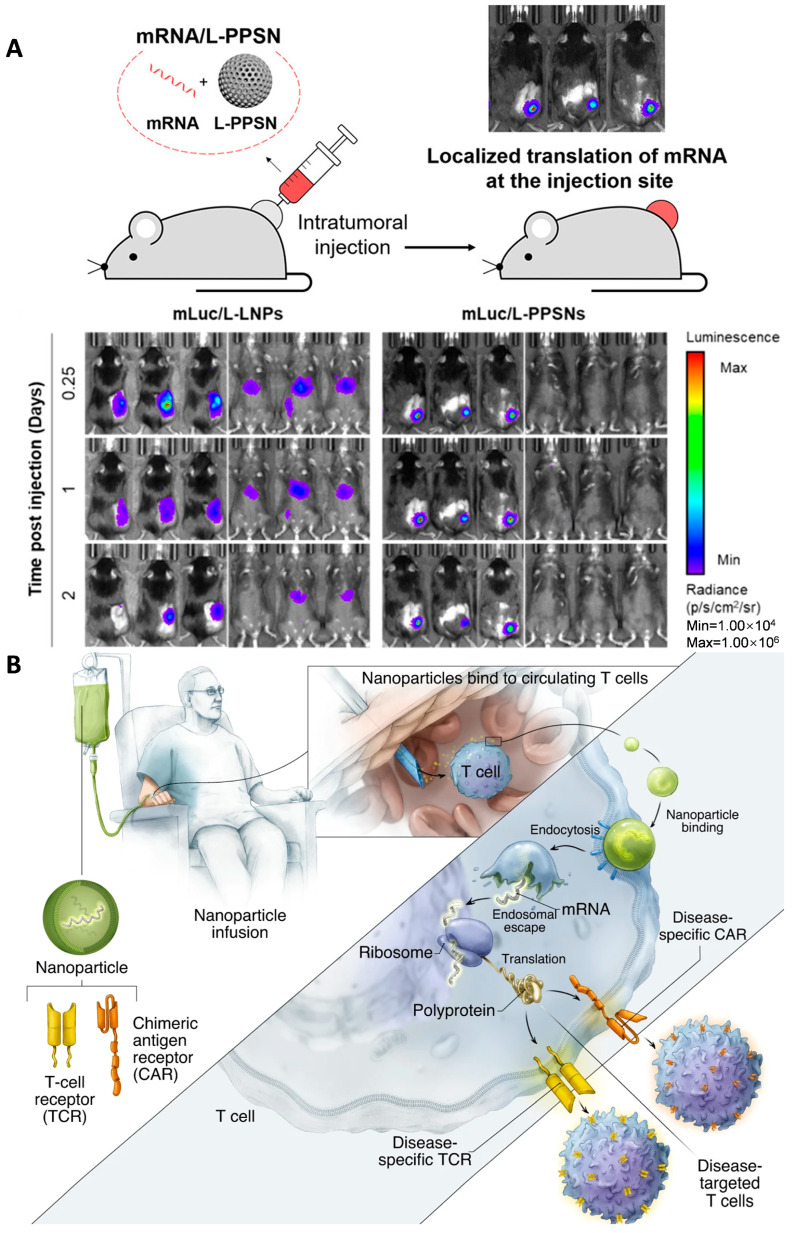
Non-viral and lipid nanomaterials for RNA delivery. (**A**) Polyethylenimine-modified Porous Silica Nanoparticle (L-PPSN) for the delivery of engineered IL-2 for antitumor immunity. Figure is adapted from [63]. (**B**) A biodegradable polymeric nanocarrier that can deliver in vitro transcribed mRNA encoding disease-specific chimeric antigen receptors (CARs) or T cell receptors (TCRs) directly to circulating T cells in vivo. Figure is adapted from [66].

Min et al. recently reported a porous silica nanoparticle-based delivery platform for localized mRNA immunotherapy against tumors (Figure 6A) [63]. Localized delivery of immunomodulatory molecules such as cytokines can stimulate anti-tumor immune responses while avoiding systemic toxicities [64]. Based on this mechanism, the authors developed a polyethyleneimine-modified porous silica nanoparticle (L-PPSN) platform to efficiently deliver cytokine mRNA, such as IL-2 mRNA, directly into the tumor microenvironment. Compared to FDA-approved LNPs, the L-PPSN platform showed superior efficiency for localized mRNA translation without any off-target expression in other organs. Intratumoral injection of L-PPSN loaded with cytokine mRNA led to high protein expression within the tumor and stimulated immunogenic cancer cell death. Combining cytokine mRNA delivery with an immune checkpoint inhibitor enhanced anti-cancer responses in multiple mouse tumor models and enabled inhibition of distant metastatic tumors. The results demonstrate the potential of the L-PPSN platform as a specific, effective, and safe delivery system for mRNA-based cancer immunotherapies that potentially overcome the limitations of systemic cytokine immunotherapy.

Current approaches for producing genetically modified T cells for cancer therapy are complex and expensive, limiting widespread application [65]. Stephan et al. explored using nanoparticles to quickly program T cells with cancer-targeting capabilities directly in the patient (Figure 6B) [66]. The authors developed biodegradable polyglutamic acid-based nanoparticles that are functionalized with T cell-targeting ligands (CD8 antibodies) and nuclear localization signals. These nanoparticles can efficiently deliver plasmid DNA encoding a chimeric antigen receptor (CAR) specific for the CD19 leukemia antigen into T cells. The CAR gene expression cassette is flanked by piggyBac transposon elements, allowing for stable chromosomal integration and long-term CAR expression in proliferating T cells. A plasmid encoding the piggyBac transposase is co-encapsulated in the nanoparticles. In vitro experiments showed that the nanoparticles can selectively bind and deliver the CAR genes into T cells, resulting in robust and persistent CAR expression on the T cell surface within 30 h. In mouse models of leukemia, administration of these CAR-expressing nanoparticles led to T cell reprogramming and potent anti-tumor activity, comparable to conventional ex vivo CAR T cell therapy. The researchers conclude that this nanoparticle platform provides a practical, broadly applicable approach to generating anti-tumor T cell immunity ‘on demand’ for cancer treatment, avoiding the complex procedures required for ex vivo genetic modification of T cells.

The CRISPR/Cas9 system holds promise for treating various diseases, but the inability to perform specific gene editing in targeted tissues and cells remains a critical bottleneck [67]. Previous delivery methods using viral vectors or non-viral nanoparticles can result in off-target effects due to the lack of cell/tissue specificity. Wang et al. constructed two macrophage-specific Cas9 expression plasmids, pM458 and pM330, by replacing the original promoters in pX330 and pX458 with the human CD68 promoter [68]. The CD68 promoter can drive specific gene expression in monocytes and macrophages. The macrophage-specific Cas9 plasmids were encapsulated in cationic lipid-assisted nanoparticles (CLANs), a type of cationic lipid-assisted PEG-b-PLGA nanoparticle, to enable in vivo delivery. The CLAN nanoparticles were able to efficiently internalize into various immune cells, including B cells, neutrophils, monocytes, and macrophages. Under the control of the CD68 promoter, the Cas9 protein was specifically expressed in monocytes and macrophages, but not in other cell types after systemic administration of the CLAN nanoparticles. The authors further encoded a guide RNA targeting the Ntn1 gene into the pM330 plasmid, forming CLANpM330/sgNtn1 [68]. This system was able to disrupt the Ntn1 gene specifically in macrophages and their precursor monocytes in vivo, reducing netrin-1 expression and improving type 2 diabetes symptoms in a mouse model. The Ntn1 gene was not disrupted in other cell types due to the macrophage-specific Cas9 expression. This study provides a strategy for achieving specific in vivo gene editing using the CRISPR/Cas9 system by combining a tissue-specific promoter with a non-viral nanoparticle delivery system. The macrophage-specific gene editing approach demonstrated the potential therapeutic application for type 2 diabetes and possibly other diseases.

Beyond these established carriers, novel alternatives such as exosome-mimicking nanovesicles and iron oxide nanoparticles have emerged [69]. Polymer nanocarriers boast high thermodynamic and kinetic stability [31], while exosome-mimicking nanovesicles excel in yield and loading efficiency [70]. Iron oxide nanoparticles, comprising Fe_3_O_4_ or Fe_2_O_3_, harness their superparamagnetism to electrostatically engage with surface-engineered cations and nucleic acids [69]. Nevertheless, the potential safety and stability concerns of these emerging vectors require further scrutiny, and the targeting issue necessitates the introduction of additional moieties. Nevertheless, these nanoscale vehicles potentially face immune and liver clearance, making the delivery of nucleic acid drugs a persistent challenge in this field.

### 3.5. Chemical Modification

**Figure 7:**
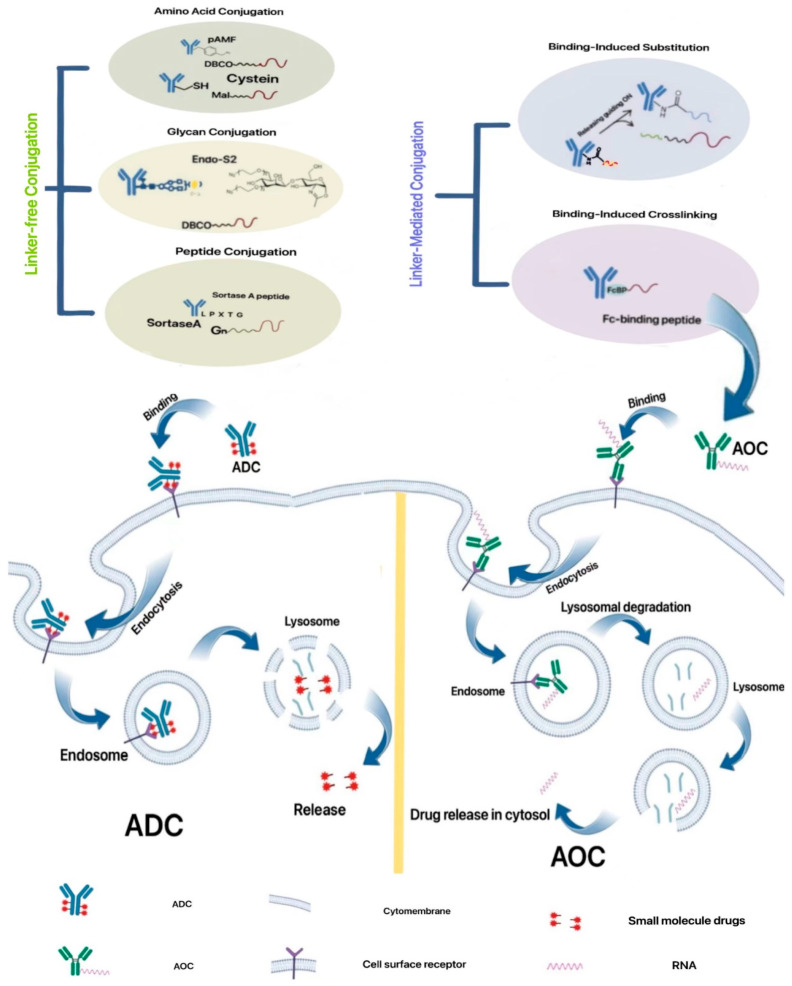
The preparation and intracellular mechanism of action of ADC drugs and antibody–oligonucleotide conjugate (AOC) drugs.

Chemical modification involves altering the RNA structure, particularly its functional groups, while preserving its chemical properties. This approach aims to enhance the in vivo stability of RNA delivery and refine its targeting capabilities. As a well-known example, N-acetylgalactosamine (GalNAc) can be covalently bound to the 3′ terminal of RNA (GalNAc–RNA) in a trivalent fashion, exhibiting a high degree of specificity and affinity for binding to the sialoglycoprotein receptor (ASGPR). This receptor, through clathrin-mediated endocytosis, facilitates the transportation of GalNAc–RNA into the cytoplasm and accumulates in the endosome. Due to the decrease in pH during endosome maturation, GalNAc dissociates from the ASGPR. Then, GalNAc and the linker are degraded to release siRNA, while the ASGPR is recovered back to the cell surface. For free siRNA molecules, most of them are still trapped in the endosome, with only a small fraction (<1%) released into the cytoplasm [71]. Once it enters the cytoplasm, siRNA will be incorporated into the RISC to exert its inhibitory function. Notably, the ASGPR is predominantly expressed on the surface of liver cells, with minimal presence in other cell types [72]. Therefore, GalNAc–RNA exhibits high selectivity for the liver and can be taken up by the liver at high levels [73]. SiRNA can exist for a long time in the endosome, while siRNA bound to the RNA-induced silencing complex (RISC) can be protected from nuclease degradation [74]. In addition, GalNAc RNA has high selectivity for the liver, which enhances the stability of siRNA and has significant advantages in treating liver diseases [75], minimizing off-target effects and reducing immunogenicity (Figure 7 and Table 1).

**Figure 8:**
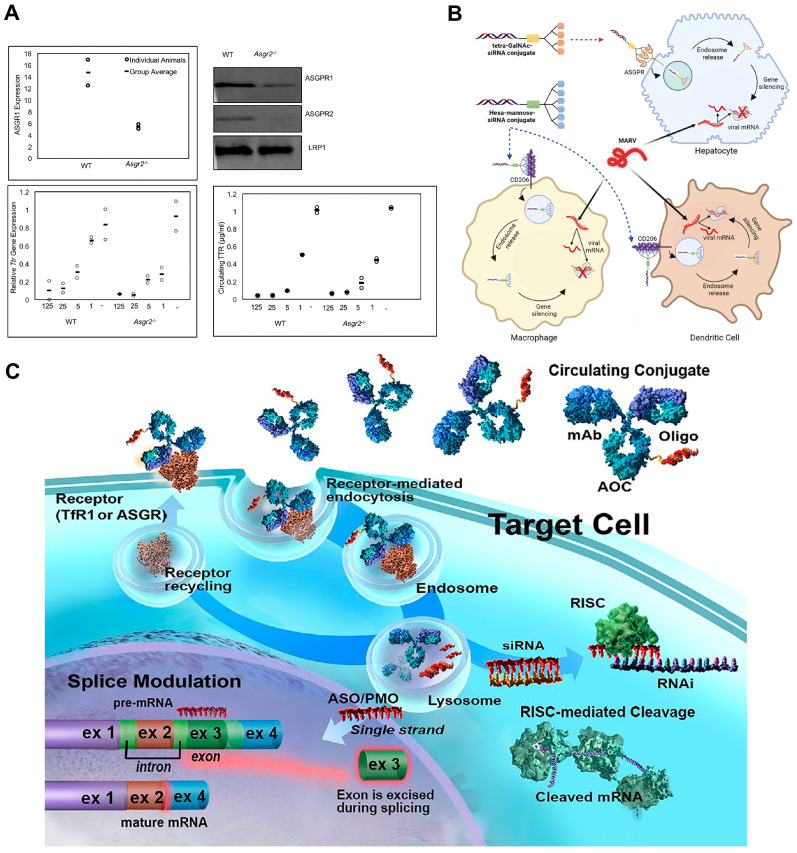
Recent clinical studies of antibody–oligonucleotide conjugates for immunotherapy. (**A**) Evaluation of the activity of GalNAc–siRNA conjugates in pre-clinical animal models with reduced asialoglycoprotein receptor expression. Figure is adapted from [76]. (**B**) Evaluation of mannose–siRNA and GalNAc–siRNA conjugates for hepatocyte infection. Figure is adapted from [78]. (**C**) Development of antibody–oligonucleotide conjugates for targeted delivery of RNA therapeutics to skeletal and cardiac muscle. Figure is adapted from [82].

GalNAc-based drugs typically require only a single injection every six months [60], indicating their extended efficacy, which is comparable to small nucleic acid drugs and even many marketed drugs. Modifications such as 2′-fluorine and 2′-methoxyl to five-membered rings of the RNA base can stabilize its structure. Mechanistically, GalNAc–RNA rapidly localizes in the liver by interacting with the ASGPR of the liver cells for clathrin-mediated endocytosis and is “stored” in the lysosome [25]. Its accumulation within the lysosome occurs at a slow rate, allowing for gradual release and sustained loading onto the RISC, thereby achieving long-term therapeutic effects. Zimmermann et al. developed GalNAc–siRNA conjugates, where a synthetic GalNAc ligand is conjugated to siRNA to enable efficient ASGPR-mediated delivery to hepatocytes (Figure 8A) [76]. To assess the impact of reduced ASGPR expression, the authors evaluated the pharmacokinetics and pharmacodynamics of GalNAc–siRNA conjugates in three pre-clinical models with decreased ASGPR levels: a. Mice with germline deletion of the ASGPR2 subunit. b. Rats with alcoholic liver disease induced by chronic ethanol exposure. c. Rats treated with the barbiturate phenobarbital. Despite a reduction in ASGPR levels of over 50% in these models, the GalNAc–siRNA conjugates retained potent activity, suggesting the remaining receptor capacity was sufficient for efficient uptake. These results support the broad application of GalNAc–siRNA technology for hepatic targeting, even in disease states where ASGPR expression may be reduced.

In addition to the four drugs that have already been commercialized and over ten drugs in the clinical trial phase, extensive research on nucleic acid drugs is available in the market [25]. Yet it remains challenging to discover compounds that rival the effectiveness of GalNAc’s chemical modification, thereby limiting its primary application in the treatment of liver diseases. Other commonly employed chemical modifications include thiophosphoric acid skeleton modification, methoxyl modification, fluoro modification, GNA modification (a chemical substance analogous to DNA or RNA), and 5′-(E)-VP modification (Table 1). However, various issues, such as being difficult to metabolize, have emerged, resulting in the current four chemically modified drugs being predominantly GalNAc–RNA drugs [26].

Currently, monoclonal antibodies exhibit high specificity but limited efficacy, while small molecule drugs have high toxicity and poor targeting. ADC drugs utilize specific linkers to connect antibodies with small molecule drugs, combining the advantages of both. By leveraging the specificity of monoclonal antibodies, ADCs enable the targeted delivery of small molecule drugs to receptor cells, enhancing therapeutic efficacy while reducing toxic side effects. Therefore, the selection of appropriate antibodies, ligands, and small molecule drugs is crucial for optimizing the effectiveness of ADC drugs [77]. Thus, Heyes et al. reported a combination treatment using two different siRNA conjugate platforms to target Marburg virus (MARV) infection (Figure 8B) [78]. MARV infection causes severe viral hemorrhagic fever with high mortality rates, and there are currently no approved vaccines or antiviral therapies [79]. The authors developed a siRNA conjugate platform using a hexavalent mannose ligand that can target macrophages and dendritic cells, which are key cellular targets of MARV infection. The mannose–siRNA conjugate enabled successful delivery and gene silencing in macrophages both in vitro and in vivo. The authors also evaluated a hepatocyte-targeting GalNAc–siRNA conjugate to expand targeting to infected liver cells. When used individually, the mannose–siRNA and GalNAc–siRNA conjugates provided limited survival benefits in MARV-infected guinea pigs. However, the combination of the two conjugates achieved up to 100% protection when administered 24 h post-infection. This novel approach of simultaneously delivering siRNA to multiple cell types relevant to MARV infection, using two different targeting ligands, provides a convenient subcutaneous route of administration for treating these dangerous pathogens. The mannose conjugate platform also has potential applications for other diseases involving macrophages and dendritic cells. This work presents the combination of two different siRNA conjugate platforms to achieve comprehensive targeting and protection against lethal MARV infection in an animal model.

Specific linkers determine the stability and payload release profiles of ADCs. Ideal linkers neither prematurely release the payload nor delay release, which could lead to ADC aggregation. Currently, chemical conjugation and enzymatic conjugation are two effective methods for linking antibody and payload components, which are also key steps in preparing ADC drugs (Figure 7). However, numerous challenges persist in the research and development of immunotherapy. The binding affinity between the antibody and antigen complex significantly impacts its internalization efficiency, with excessively high affinity potentially hindering penetration into solid tumors. Therefore, achieving an optimal affinity level is crucial to balancing rapid absorption and anticancer efficacy [80]. Furthermore, the bulky nature of antibodies poses challenges in penetrating capillary and tumor tissue matrices. Consequently, miniaturizing antibodies and managing the size of the chosen small molecule drugs is essential. Nevertheless, miniaturized antibodies often exhibit shorter half-lives, necessitating a delicate balance [81]. Additionally, the intricate pharmacokinetics and limited payload capacity of immunotherapy present obstacles that require further refinement.

By introducing the aforementioned two drug types, researchers aim to combine their respective strengths, yielding novel therapeutics that offer high targeting precision, minimal toxic side effects, compact size, robust stability, and superior efficacy. As early as 2005, Judy Lieberman of Harvard University pioneered the conjugation of siRNA targeting H1V-1 capsid protein mRNA to the antibody FAB fragment using protamine as a linker, laying the foundation for antibody–oligonucleotide conjugates (AOCs) [82]. In 2019, the world witnessed the commencement of clinical trials for AOC 1001, the first AOC drug to enter Phase I, marking a significant milestone in the development of this drug class [83].

AOC drugs employ a strategy akin to ADC drugs, substituting siRNA or ASO for small molecule drugs. So far, there are numerous methods of conjugation which can be divided into two ways, namely linker-free conjugation and linker-mediated conjugation. To begin with, assuming there is no linker, the antibody and RNA can be conjugated by powerful covalent bonds, such as amino acid, glycan, and peptide. Some representative compounds might be cysteine with sulfhydryl, pAMF with dibenzoazacyclooctyne (DBCO), Endo-S2 with DBCO, and Sortase A peptide, respectively (Figure 7). Moreover, there are diverse linkers that can play a vital role in conjugation. With binding-induced substitution, double-stranded RNA is ligated with amide bonds, after which a single strand is released. Furthermore, for the sake of binding-induced crosslinking, the Fc-binding peptide is a stable selective [29]. This approach endows nucleic acid drugs with the same targeting capabilities as immunotherapy while significantly enhancing the stability of small nucleic acids.

However, the choice of conjugation method plays a crucial role in determining the pharmacokinetics and efficacy of this drug class [84]. Currently, various coupling modes are in use, including charge affinity, streptavidin-biotin binding, direct coupling, and base complementary pairing. These different coupling mechanisms exert a profound influence on the absorption, distribution, metabolism, and excretion (ADME) properties.

Similar to ADC drugs, AOC drugs are still in the nascent stages of research and development. Given that they integrate two emerging therapeutic modalities—nucleic acid drugs and immunotherapy—the ADME, efficacy, and safety considerations are particularly intricate and lack ample clinical data [85]. Despite their combined advantages, numerous factors must be taken into account, and their intricate pharmacokinetics can only be gradually elucidated through extensive calculations, experiments, and clinical applications. Recently, Levin et al. generated AOCs using anti-TfR1 (transferrin receptor 1) monoclonal antibodies conjugated to different types of oligonucleotides, namely siRNA, antisense oligonucleotides, and morpholine oligonucleotides (PMOs) (Figure 8C) [82]. In mice, the TfR1-targeted AOCs achieved over 15-fold higher accumulation in muscle tissue compared to unconjugated oligonucleotides. A single dose of a TfR1-targeted siRNA AOC against Ssb mRNA resulted in over 75% mRNA reduction in both mice and non-human primates, with the silencing effect being highest in striated muscle. In mice, the EC50 for Ssb mRNA reduction in skeletal muscle was over 75-fold lower than in systemic tissues, indicating the targeted delivery to muscle. Oligonucleotides conjugated to control antibodies or cholesterol showed much lower potency compared to the TfR1-targeted AOCs. The pharmacokinetic and pharmacodynamic properties of the AOCs demonstrated that the mRNA silencing activity is primarily driven by the receptor-mediated delivery to striated muscle. The results were consistent across different oligonucleotide modalities (siRNA, ASOs, and PMOs) in mice, and the AOC properties translated to higher species (non-human primates). This study demonstrates the efficacy of using TfR1-targeted AOCs to selectively deliver various oligonucleotide therapeutics to skeletal and cardiac muscle, providing a promising approach to expand the therapeutic potential of RNA-based drugs beyond the liver.

## 4. Conclusions

In the previous section, we reviewed RNA-based immunotherapy and its delivery system. The delivery system mainly includes carrier transportation and chemical modification. In addition, the principles, advantages, and limitations of various delivery methods were compared. Immunotherapy stands as a preeminent treatment modality, with ADC drugs harnessing the precision of monoclonal antibodies to deliver small molecule drugs. This fusion has sparked the cross-fertilization of immunotherapy and nucleic acid drugs, precipitating a convergence that has given rise to AOC drugs—an evolution of ADC drugs. The advent of nucleic acid drugs opens up a vast array of promising targets, addressing the challenges posed by diverse drug targets. Coupled with their benefits of superior efficacy and extended therapeutic effect, these drugs have gained significant traction in recent research. However, nucleic acids face several challenges, including limited half-life, poor stability, high immunogenicity, and challenges in precise delivery and cellular entry. Overcoming these obstacles hinges on the development of an optimal delivery mechanism. Currently, the delivery of nucleic acid drugs is primarily achieved through chemical modification and the use of delivery carriers. In recent years, chemically modified delivery materials have achieved remarkable success, with GalNAc–RNA drugs effectively enhancing RNA stability and compound targeting.

Furthermore, inorganic nanocarriers such as SSNs, MSNs, gold nanoparticles, quantum dots, and iron oxide nanoparticles, as well as other platforms such as VLPs, polymer-carriers, and exosome-mimetic nanovesicles, have emerged as novel research directions. Even existing drugs have achieved efficacy spans of up to six months. Notably, many marketed drugs employ GalNAc chemical modification methods and AAV virus vectors. However, the specificity of GalNAc confines its application to liver diseases, while immune recognition can lead to resistance against viral vectors, limiting their long-term use.

In conclusion, there is an urgent need to explore novel delivery methods, building upon the foundations of chemical modification strategies. AOC drugs boast remarkable targeting capabilities and significantly bolster RNA stability, often achieved either directly or via linker-mediated conjugations. To enhance the therapeutic duration of AOC drugs, future research can explore avenues such as in vitro B cell culture techniques, chemical modifications that modulate entry and release kinetics, click chemistry and biological orthogonal reactions, and even computer-assisted drug design or AI technology to optimize drug structure, predict potential drug targets, and assist in the development of pharmacokinetic models. This interdisciplinary integration promises to accelerate the AOC drug development process, enhance drug efficacy and safety, and broaden the horizons for AOC drugs, offering significant benefits for future disease treatments and the R&D of nucleic acid drugs and their delivery systems.

Although AOC drugs hold significant promise, the instability of RNA drugs in vivo and their limited duration of action cannot be overlooked. It is possible to utilize novel perspectives and outlooks of immunotherapy combined with click chemistry to address the short-term effects of AOC drugs. Currently, the primary research focus of AOC drugs lies in modulating pharmacokinetic effects through optimized coupling modes. Despite the promising research and development prospects associated with AOC drugs, which are attributed to their strong targeting, high stability, and minimal toxic side effects, it is essential to acknowledge the inherent challenges posed by the in vivo instability of RNA drugs and their limited duration of action.

Presently, there have been numerous studies and successful cases utilizing click chemistry to prepare AOC drugs. For instance, antibodies containing alkynes and RNAs containing azides rapidly combine to produce AOC drugs with triazole as the linker. Simultaneously, RNA-based click chemistry has also been applied to various animal studies [82]. Therefore, we believe that combining the methods of ex vivo cultivation of T or B cells and click chemistry for preparing AOC drugs may address the issues of short duration and short half-life associated with AOC drugs.

## Figures and Tables

**Table 1:** The principles, advantages, and disadvantages of the different delivery methods of RNA.

Name	Principle	Advantages	limitation	Reference
GalNAc	GalNAc binds highly specifically to the ASGPR receptor, allowing rapid targeting of the liver and entry into cells in the form of endocytosis.	Low off-target effect, low immunogenicity, improved stability, strong targeting, long duration of efficacy, no cytotoxicity, high efficacy.	Targeting only the liver has many limitations, and the targeting of normal liver cells is stronger than that of diseased liver cells	[25,26]
Thiophosphoric acid skeleton modification, methoxyl modification, fluoro modification	By changing the structure of the RNA, the stability of the RNA is increased while ensuring biological affinity, so that it can have more time to reach the target site with blood circulation.	Enhancing the stability of RNA, not easily degraded, and enhancing the half-life of RNA.	It affects the overall chemical properties of nucleic acids and even limits their function, producing different isomers and changing the function.	[27]
GNA	RNA oligomers containing GNA residues have been shown to form double strands with DNA and RNA to enhance stability while increasing their targeting.	Improving the thermal stability of RNA, reducing off-target effects, and easy chemical synthesis. It has significant hybridization characteristics, improving the therapeutic index of RNA.	It does not exist in nature so there may be certain safety issues.	[28]
Antibody (AOC)	Through the binding of monoclonal antibodies corresponding to the receptors on the surface of the target cells, RNA is rapidly delivered to the target site through the targeting of the antibody.	Strong targeting, low-toxicity side effects, small size, high stability, high efficacy.	The pharmacokinetics are complex, the efficacy is short, and the monoclonal antibody is degraded after endocytosis.	[29]
Lipid Nanoparticles (LNPs) and Liposomes	RNA is encapsulated by LNPs to avoid disease clearance while enhancing its stability, enabling targeted delivery through altered permeability in the tumor microenvironment or modification on the lipid surface.	Strongly lipophilic, enhancing RNA stability, difficult immune clearance, easy to enter the cell, simple preparation, easy metabolism.	The addition of PEG resulted in cytotoxicity, serious off-target effects, short storage time, and harsh transport conditions.	[30]
Viral Vector	RNA is placed into an AAV vector to mimic the process of virus invasion into cells, and cytolysis is achieved by binding the viral protein to the receptor on the surface of the target cell	Strong targeting, enhanced RNA stability, no cytotoxicity, and strong self-stability.	Easily cleared by the immune system and will lead to drug resistance, leading to reduced efficacy.	[31,32,33]
Delivery Carrier Utilizing Inorganic Materials	RNA is stored in the pores of inorganic materials to form a special solid–liquid interface, and the stability of RNA in solid media is greatly increased. Specific functional groups are added to the surface of inorganic materials to achieve targeted delivery.	Enhanced RNA stability, difficult immune clearance, flexible design size and shape, easy preparation, preparation process pollution is small, low cost.	There are certain off-target effects, security problems, and low targeting.	[34,35]
Quantum Dot	The delivery of quantum dots is still in the theoretical stage, and the target is achieved by depositing RNA in quantum dots to achieve point delivery of RNA.	Strong stability, scalable application to diagnosis, and good efficacy.	The load is limited and PEG has a certain toxicity.	[36]
Polymer nanocarriers	Enhancement of RNA stability is achieved by means of multimers, and targeting is achieved by modifying specific structures.	High thermodynamic and kinetic stability, simple and scalable synthesis, diverse structure, and high transfection rate.	Endosomal escape disorder, metabolic problems, and potential toxicity after carrier delivery.	[37]

## Abbreviations

AAV	adeno-associated virus
ADCs	antibody-Drug Conjugates
ADME	absorption, distribution, metabolism, and excretion
AOCs	antibody-oligonucleotide conjugates
ASGPR	sialoglycoprotein receptor
ASO	antisense oligonucleotide
BCR	B-cell receptor
CAR	chimeric antigen receptors
ChAd68	chimpanzee adenovirus
CLAN	cationic lipid-assisted nanoparticles
ctDNA	circulating tumor DNA
CTL	cytotoxic T lymphocytes
DBCO	dibenzoazacyclooctyne
DC	dendritic cell
EMA	European Medicines Agency
FDA	Food and Drug Administration
GalNAc	N-acetylgalactosamine
hATTR	hereditary transthyretin amyloidosis
HCC	hepatocellular carcinoma
HIV	human immunodeficiency virus
HO1	heme oxygenase-1
ICD	immunogenic cell death
ICIs	immune checkpoint inhibitors
IL-12	interleukin-12
LNPs	Lipid Nanoparticles
L-PPSN	polyethylenimine-modified porous silica nanoparticle
MARV	Marburg virus
MDSCs	Myeloid-derived suppressor cells
mRNA	messenger RNA
MSNs/MSNPs	mesoporous silica nanoparticles
MSS-CRC	microsatellite-stable colorectal cancer
PCL	poly(ε-caprolactone)
PD-L1	programmed cell death protein 1
PEG	polyethylene glycol
PEI	polyethyleneimine
PGs	poly(glycerols)
PHEMA	poly(2-hydroxyethyl methacrylate)
PHPMA	poly(hydroxypropyl methacrylate)
PLGA	poly(lactic-co-glycolic acid)
PMDA	Pharmaceuticals and Medical Devices Agency
PMO	morpholine oligonucleotide
POX	poly(oxazolines)
samRNA	self-amplifying RNA
siRNA	small interference RNA
SIV	simian immunodeficiency virus
SSNs	spiked silica nanoparticle
TAMs	tumor-associated macrophages
TCRs	T cell receptors
TDLN	tumor draining lymph node
TfR1	transferrin receptor 1
TLR	Toll-like receptors
TME	tumor microenvironment
TTR	transthyretin
VLP	virus-like particle
